# CT radiomics for survival risk stratification in resectable colorectal liver metastases: a multi-centre study

**DOI:** 10.1038/s41598-026-48659-0

**Published:** 2026-06-24

**Authors:** Areeb Mian, Robert Young, Adil S. Lakha, Ioannis Leptidis, Robert P. Jones, Alex Gordon-Weeks

**Affiliations:** 1 Nuffield Department of Surgical Sciences, University of Oxford, John Radcliffe Hospital, Oxford, OX3 9DU UK; 2 Department of Hepato-Pancreato-Biliary (HPB) Surgery, Oxford University Hospitals NHS Foundation Trust, Oxford, OX3 9DU UK; 3 Human Liver Research Facility, University of Liverpool, Liverpool, UK; 4 Department of Hepatobiliary Surgery, Liverpool University Hospitals NHS Foundation Trust, Liverpool, UK; 5 Department of Molecular and Clinical Cancer Medicine, Institute of Systems, Molecular and Integrative Biology, University of Liverpool, Liverpool, UK; 6 Department of Pharmacology, Institute of Systems, Molecular and Integrative Biology, University of Liverpool, Liverpool, UK

**Keywords:** Imaging biomarkers, Tumour heterogeneity, Machine learning, Image based phenotyping, Precision surgical oncology, Cancer, Gastroenterology, Oncology

## Abstract

Outcomes following resection of colorectal liver metastases (CRLM) remain heterogeneous, and there is a need for preoperative tools that support survival risk stratification and clinical decision making. CT based radiomics offers a non invasive approach to quantify tumour and liver characteristics using routinely acquired imaging. Here we report the largest multicentre study evaluating the prognostic utility of preoperative CT radiomics in patients undergoing resection of colorectal liver metastases. Patients undergoing curative-intent CRLM resection were retrospectively identified from two UK centres. Radiomic features were extracted from portal-venous CT scans of metastases and background liver. Feature selection techniques were applied. Machine learning models incorporating clinical and/or radiomic variables were trained to predict 3-year mortality. A Radiomic Risk Score (RRS) was derived from six radiomic features and evaluated using Cox regression and Kaplan–Meier analysis in an independent validation cohort. A total of 959 metastases from 399 patients were analysed. Six radiomic features (one morphological, one first-order, and four textural) were retained. Radiomics alone demonstrated moderate discrimination for 3-year mortality (AUC 0.77, 95% CI 0.68–0.85), comparable to clinical models (AUC 0.82, 95% CI 0.75–0.86). Combined clinical–radiomic models demonstrated modest but consistent improvement in performance (random forest AUC 0.83, 95% CI 0.78–0.88). At both 2-year and 3-year timepoints, formal comparison using the DeLong test demonstrated no statistically significant difference between radiomics and clinical models. The RRS stratified patients into high- and low-risk groups with significantly different survival in both the training cohort (HR 2.71, 95% CI 1.86–3.96, *p* < 0.001; median OS 32 vs. 53 months) and validation cohort (HR 2.01, 95% CI 1.14–3.54, *p* < 0.001; median OS 24 vs. 38 months). Multivariable analysis confirmed the RRS as an independent prognostic factor (HR 2.91, 95% CI 1.93–4.39, *p* < 0.001). Preoperative CT radiomics provides complementary prognostic information in patients undergoing resection of colorectal liver metastases. While radiomics alone does not outperform clinical variables for fixed-horizon prediction, it enables robust survival risk stratification and improves combined model performance. Radiomics could be considered as an adjunct to, rather than a replacement for, established clinical assessment in CRLM.

**Supplementary Information:**

The online version contains supplementary material available at 10.1038/s41598-026-48659-0.

## Introduction

Survival outcomes following surgical resection for colorectal liver metastases (CRLM) have improved significantly over the past two decades, driven by advances in systemic chemotherapy, perioperative care, and refinements in surgical technique^1–3^. Resection is now offered to patients with higher tumour burden provided that an adequate future liver remnant can be preserved, reflecting a shift away from historical contraindications^2,4^. Despite this, postoperative outcomes remain heterogeneous, with recurrence occurring in more than half of patients^5–10^. Existing clinical risk scores, provide only modest prognostic accuracy, underscoring the need for additional tools to refine patient selection and guide personalised management strategies^11^.

There is therefore a need for non-invasive biomarkers that are widely accessible, easily integrated into clinical workflows, and applicable using existing standard-of-care modalities. In current practice, contrast-enhanced computed tomography (CT) is the cornerstone imaging modality for staging and surgical planning in CRLM, owing to its wide availability and standardised reproducibility. But conventional CT assessment remains largely qualitative, focusing on the size, number, and distribution of metastases, providing limited insight into tumour biology^12^.

Radiomics generates quantitative data from radiological images, offering the potential to transform imaging evaluation from subjective visual estimation into an objective, reproducible biomarker science^13^. This enables non-invasive phenotyping of tumours, capturing biological attributes and heterogeneity imperceptible to the naked eye^13,14^. Radiomics has shown promise across cancer types in early diagnosis^15–17^, prognostication^18–20^ and therapy response prediction^21,22^. However, in CRLM, existing radiomics studies have been limited to small, single-centre retrospective cohorts, lacking sufficient validation and at risk of overfitting. Many such studies report very high discrimination metrics, which likely overestimate the true accuracy. This is frequently driven by the inclusion of a large number of radiomic features relative to sample size, coupled with the absence of external validation, limiting assessment of real-world generalisability across institutions, scanners, and imaging protocols^23–26^.

In this study, we address these limitations by developing and validating a CT-based radiomics approach using portal venous phase imaging from two UK hepatobiliary centres. We evaluate the performance of radiomics relative to established clinical factors for prediction of 3-year and 2-year mortality and assess its prognostic value using a time-to-event framework in an independent real-world validation cohort. By explicitly distinguishing between binary outcome prediction and survival risk stratification, this work aims to provide a realistic assessment of the current clinical utility of CT-based radiomics and to clarify directions for future work.

## Methods

### Participating centres

Patients undergoing curative-intent liver resection for colorectal liver metastases (CRLM) were identified at Aintree University Hospital, Liverpool, and the Churchill Hospital, Oxford. Data were obtained from prospectively maintained institutional databases covering the period 01/01/2015 to 31/12/2019 at both centres. This study was conducted in accordance with the Declaration of Helsinki and relevant institutional guidelines and regulations. Ethical approval was obtained at each participating centre (North West Research Ethics Committee [NW REC 15/NW/0477] and local audit approval [03-7089]), alongside overarching approval from the South Central Oxford C Research Ethics Committee (REC 25/SC/0150). Given the retrospective nature of the study and use of anonymised data, the requirement for informed consent was waived by the approving Research Ethics Committees.

### Inclusion/exclusion criteria

Patients within the Liverpool database included all those undergoing any form of CRLM resection with a background of positive primary colorectal cancer histology. The Oxford database contained patients undergoing major hepatectomy (≥3 vascular segments) as reported previously^27^.

Exclusion criteria were previous hepatectomy, non-colorectal liver metastasis histopathology, inaccessible pre-treatment CT imaging, incomplete clinical prognostic data, or inadequate image quality. Inadequate image quality was defined as inability to clearly identify the metastasis, absence of a portal venous phase acquisition, or slice thickness outside the range of 1 to 5 mm. Scanner manufacturer and model were not used as exclusion criteria. Reporting followed the TRIPOD-AI guidelines^28^.

### Image processing and feature extraction

**Fig. 1:**
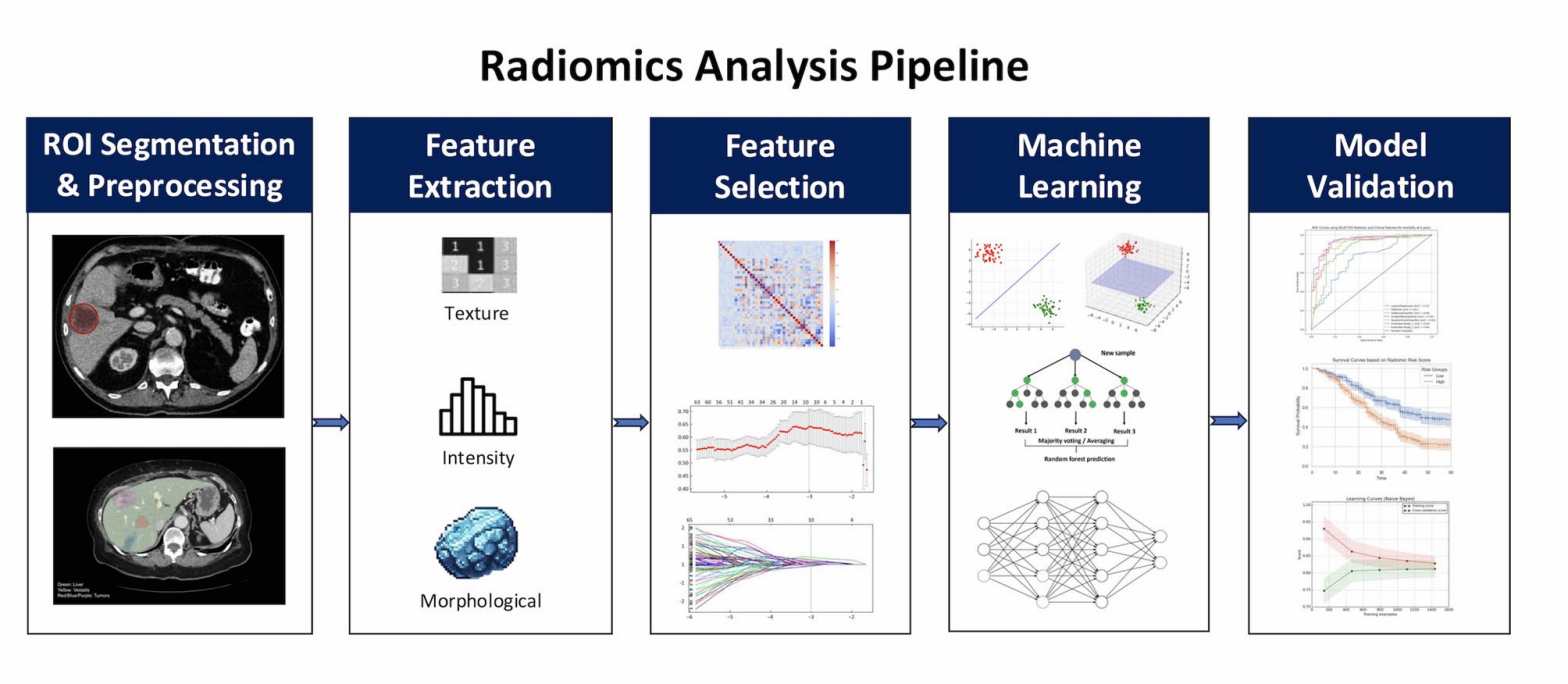
Radiomics analysis pipeline for predictive modeling and survival analysis: Regions of interest (ROIs) were manually segmented on tumour and background liver, followed by feature extraction (first-order, textural, and morphological descriptors). Machine learning algorithms (logistic regression, random forest, and ensemble models) were then trained. Model performance was evaluated using AUC, F1-score, and accuracy, and prognostic value was assessed with Kaplan-Meier survival analysis.

Regions of interest (ROIs) were generated by two authors (AG-W, RY). Images were processed using LifeX software^29^. ROIs were developed in 2D at the centre of each CRLM in the axial plane and from the background liver (Fig. S1A, B). Radiologically demonstrated CRLM were defined as hypoattenuating lesions in the portal venous phase CT with morphological features typical of metastases and identified in a multidisciplinary team meeting. Voxel size was normalised to 1.0/1.0/1.0. Radiomic features were extracted exclusively from preoperative, pre-treatment portal venous phase CT scans. Figure 1 highlights the radiomics analysis pipeline.

### Feature pre-processing and selection

A total of 267 radiomic features (first-order, texture, and morphological) were extracted. After excluding non-Image Biomarker Standardisation Initiative (IBSI)-compliant features, 207 proceeded to analysis^30^.

Highly correlated features (Pearson |r| > 0.75) were removed, leaving 132 independent features (Figure S2A-B). SelectKBest filtering identified the top 35 prognostic features^31^, which were then reduced using least absolute shrinkage and selection operator (LASSO) Cox regression with 5-fold cross-validation, yielding nine radiomics feature candidates (Figure S2C). Finally, univariate Cox proportional hazards regression in the training cohort identified six features that were individually significantly associated with overall survival (*p* < 0.05). These six features were incorporated into the multivariate Cox model, which was then used to generate a radiomic risk score (RRS) (Supplementary Table S2) as described below and in Figure S4.

### Outcome measures

The clinical endpoint was overall survival (OS), defined as the time from surgery to the last follow-up or death from any cause. For machine learning models, survival was evaluated as a binary outcome at 3 years with patients classified as alive or deceased at this time point. This cutoff was chosen as a clinically meaningful survival milestone and ensured a balanced distribution of events for model training. An additional analysis was performed at 2 years.

For the radiomic risk score, OS was analysed as a time-to-event outcome across the full follow-up period. Patients were stratified into high- and low-risk groups using the training-set median radiomics risk score, and survival differences between groups were evaluated using Kaplan–Meier analysis and Cox proportional hazards regression.

### Binary mortality prediction models

To evaluate the incremental prognostic value of radiomics, three model classes were prespecified: clinical only, radiomics only, and combined clinical radiomic models. The clinical feature set comprised ten routinely available variables selected a priori based on established prognostic relevance in CRLM and consistent availability across both centres. These included age, primary tumour nodal status, synchronous presentation, primary tumour sidedness, number of liver metastases, bilobar liver involvement, neoadjuvant chemotherapy, largest tumour size (Fong cut-off of 5 cm), resection margin status, and presence of lung metastases. These variables were used unchanged in all clinical only models and formed the clinical component of the combined models.

Three supervised machine learning classifiers were trained which included logistic regression (linear learner), random forest (parallel tree-based learner), and gradient boosting (sequential tree-based learner). Two ensemble approaches were also implemented: a Voting Classifier, which aggregated class probabilities from individual models, and a Stacking Classifier, which combined model predictions via a logistic regression meta-model^32^. Hyperparameters were optimised using grid search within a 10-fold cross-validation framework in the training cohort. Model performance was evaluated in the independent test set, with bootstrapping applied to generate confidence intervals. Receiver operating characteristic (ROC) curves were generated and area under the curve (AUC), accuracy, and F1 scores were reported at the 3-year time point.

### Radiomic risk score

A multivariable Cox proportional hazards model was constructed using the six radiomic features identified in the training cohort (Table S2). The regression coefficients from this model were combined to generate a Radiomic Risk Score (RRS) for each patient, calculated as a linear combination (Supplementary Fig. S4). The RRS was computed as a continuous score representing the weighted sum of the selected radiomic features, where each feature was multiplied by its corresponding Cox regression coefficient. This approach reflects the combined multivariable contribution of all features rather than the effect of individual features in isolation. The median RRS in the training cohort was used as the cut-off to categorise patients into high- and low-risk groups. This threshold was then applied to the independent test cohort, where patients were similarly stratified. Kaplan–Meier survival curves were generated for both groups in the training and test sets, and survival differences were evaluated using the log-rank test. The prognostic significance of the RRS was further quantified by hazard ratios (HR) and 95% confidence intervals derived from the Cox model. Feature coefficients, hazard ratios, and p-values are reported in Supplementary Table S2.

### Statistical analysis

The dataset was split at the patient level into training (70%) and test (30%) cohorts, with sampling performed to preserve proportional representation of patients from Oxford and Liverpool in each split. The test set remained completely independent and was not accessed during feature selection or model training, being used only for final evaluation. To reduce the influence of outliers, feature scaling was applied using the RobustScaler method, which standardises features based on the median and interquartile range (IQR)^33^. Continuous clinical variables were compared using Student’s t-test, and categorical variables using Fisher’s exact test. Survival estimates were generated using the Kaplan–Meier method, with differences between groups assessed by the log-rank test. A two-sided p-value < 0.05 was considered statistically significant. All machine learning, feature preprocessing, and survival analyses were implemented in Python using open-source libraries, including scikit-learn, scikit-survival, and lifelines^33,34^. AUCs for the best-performing models in each feature set were compared using the DeLong test.

## Results

**Fig. 2:**
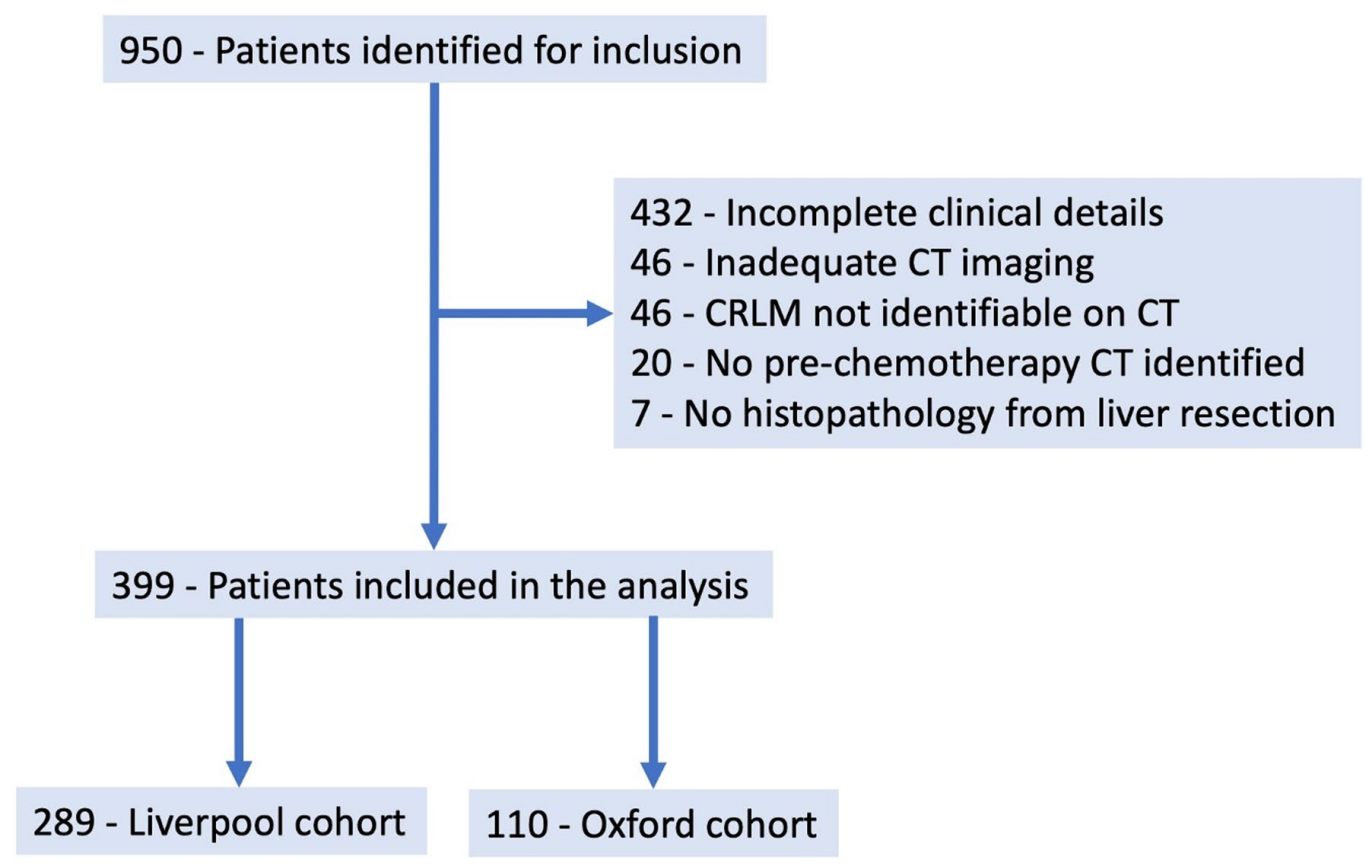
Patient recruitment flow chart and exclusion criteria. This flowchart illustrates the selection process for the study cohort. Out of 950 patients initially identified, 551 were excluded due to various criteria: 432 with incomplete clinical details, 46 with inadequate CT imaging, 46 where CRLM was not identifiable on CT, 20 without a pre-chemotherapy CT scan, and 7 without histopathology from liver resection. A total of 399 patients were included in the final analysis, with 289 from the Liverpool cohort and 110 from the Oxford cohort. The exclusion of patients without a pre-chemotherapy CT scan was necessary to ensure consistency in the baseline imaging data. This ensured an accurate, untreated depiction of the liver metastasis, allowing for a standardised assessment of radiomic features. Including patients with post-chemotherapy scans could introduce variability due to treatment effects, which may alter the appearance and texture of the metastasis on imaging, thereby impacting the accuracy of radiomic feature extraction and subsequent analysis.

The study flow and exclusions are shown in Fig. 2. Overall, 399 patients with 959 colorectal liver metastases underwent curative intent resection, with a median of 2 metastases per patient. Bilobar disease was present in 40.9%, synchronous presentation in 62.9%, and synchronous lung metastases in 16.0%. Major hepatectomy was performed in 65.9% of patients, with 17.0% managed laparoscopically. KRAS mutation status was available only in a subset of patients (Table 1).

**Table 1:** Clinical demographics of included patients.

Number of patients	Value	Missing data
	399	*n*/a
Age^b^ (years)	64 (57-71)	0
Male (%)	275 (68.9)	0
BMI^a^	27.2 ± 0.4	8 (2.5)
ASA	2 ±0.03	0 (0)
Total metastases	959	n/a
Metastases per patient^b^	2 (1-3)	0 (0)
Bi-lobar liver metastases (%)	163 (40.9)	0 (0)
Synchronous primary (%)	250 (62.9)	0 (0)
Node positive primary (%)	275 (68.9)	0 (0)
Left-sided primary (%)	318 (79.7)	0 (0)
Synchronous lung metastasis (%)	64 (16)	0 (0)
KRAS mutation (%)	80 (41.2)	205 (64.4)
Largest tumour size (mm)	30 (16–45)	3
R1 Margin (%)	120 (30.1%)	
Operation (%)		0 (0)
Trisectionectomy	36 (9)	
Hemi-hepatectomy	87 (21.8)	
Segmental resection	177 (44.4)	
Multiple wedges	52 (13)	
Single wedge	24 (6)	
ALPPS	7 (1.8)	
Other	22 (5.5)	
Major hepatectomy (%)	263 (65.9)	0
Intra-operative blood loss (ml)	759 ± 65	48 (12.0)
Laparoscopic (%)	68 (17)	0 (0)
Length of stay (days)^b^	5 (4-9)	12 (3.8)
Major morbidity (%)	41 (10.3)	16 (5.0)
30-day mortality (%)	3 (0.8)	0 (0)
90-day mortality (%)	7 (1.8)	0 (0)
Median follow-up (months)	48	0 (0)
Median overall survival (months)	54	n/a

^a^Mean ±SE, ^b^Median (IQR),

### Radiomic feature selection

Feature reduction identified six radiomic features significantly associated with OS in the training cohort: MET_MORPHOLOGICAL_Compactness2 (IBSI: BQWJ), MET_INTENSITY-HISTOGRAM_MaximumGreyLevel (IBSI:3NCY), MET_GLCM_Correlation (IBSI: NI2N), NOR_GLRLM_ShortRunHighGreyLevelEmphasis (IBSI: GD3A), NOR_NGTDM_Strength (IBSI:1 × 9X), and NOR_GLSZM_ZoneSizeEntropy (IBSI: GU8N). These comprised one morphological, one first-order histogram, and four textural features derived from both tumour and background liver regions (Table S2). These features are described in further detail in supplementary tables S3 and S4.

### 3-year and 2-year mortality prediction

**Fig. 3:**
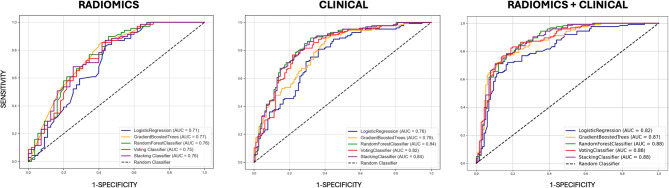
Radiomics machine learning model performances. Receiver-operating characteristics of the indicated models (colour coded with key inset for each curve) for binary mortality outcomes at 3 years of the clinical, radiomics, and combination model.

Models incorporating clinical parameters performed well for prediction of 3-year mortality (random forest AUC 0.82, 95% CI 0.75–0.86). Radiomics-only models showed similar performance, with gradient boosting achieving the highest AUC of 0.77 (95% CI 0.68–0.85). Combined clinical-radiomic models achieved the best overall performance, with the random forest model performing strongest (AUC 0.83, 95% CI 0.78–0.88) (Supplementary Table S1 and Fig. 3).

When evaluated at 2 years, radiomics-based models demonstrated improved performance compared with 3-year prediction, with AUCs up to 0.87 (95% CI 0.82–0.92). Clinical models achieved AUCs up to 0.84 (95% CI 0.78–0.90), and combined models reached AUCs of 0.90 (95% CI 0.87–0.92) (Supplementary Table S1 and Fig. S6).

To formally compare model discrimination, AUCs were compared using the DeLong test for correlated ROC curves (Figure S7). There was no statistically significant difference between radiomics-only and clinical-only models for prediction of 3-year mortality (*p* = 0.18), consistent with overlapping confidence intervals. Combined clinical–radiomic models demonstrated improved discrimination compared with radiomics-only models (*p* = 0.04), and a trend towards improved performance compared with clinical-only models (*p* = 0.06), although this did not reach statistical significance. Similar findings were observed in the 2-year mortality analysis, where no statistically significant difference was identified between radiomics and clinical models (*p* = 0.14), while combined models demonstrated improved discrimination compared with radiomics-only models (*p* = 0.03) and clinical models (*p* = 0.02).

### Radiomic Risk Score survival analysis

**Fig. 4:**
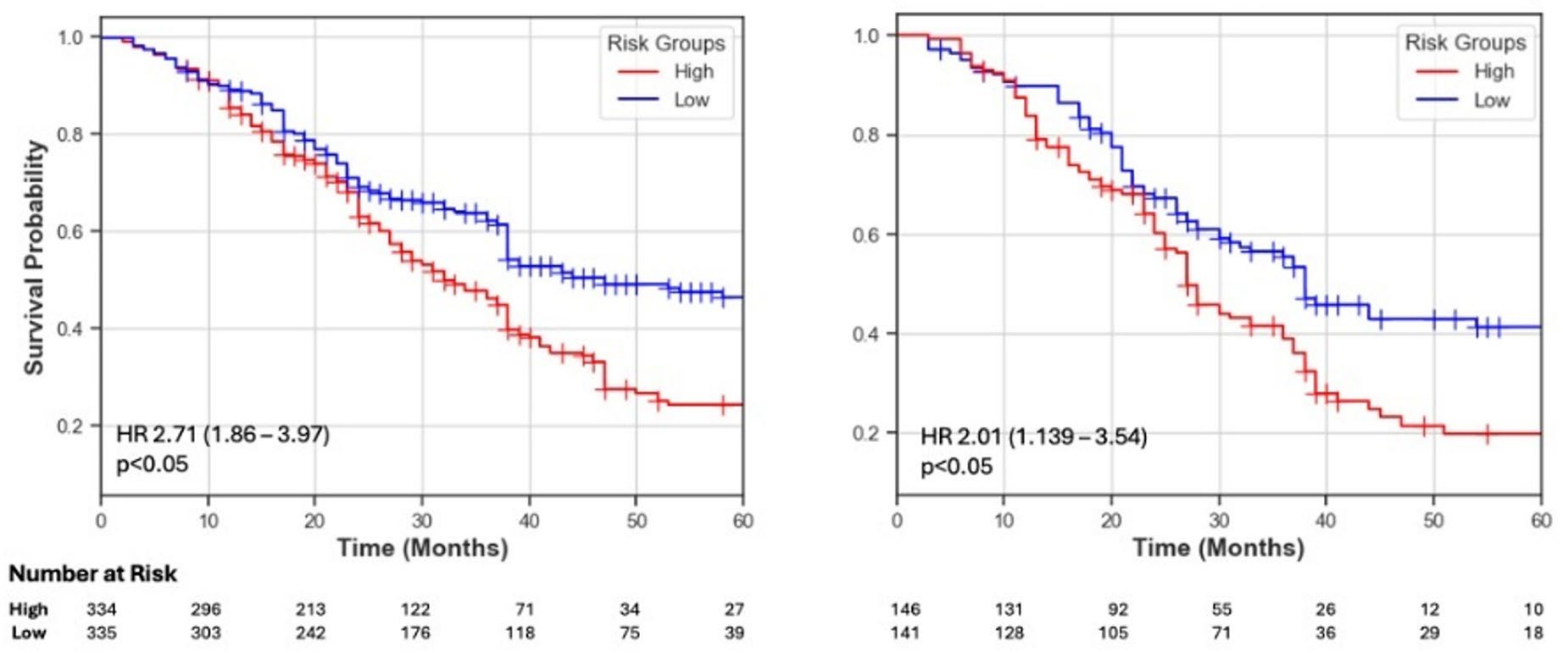
Kaplan–Meier survival analysis by Radiomic Risk Score. Patients were stratified into high- and low-risk groups using the median risk score from the training cohort. Kaplan–Meier curves are shown for (i) the training set and (ii) the independent validation set. In both cohorts, the log-rank test demonstrated a statistically significant difference in overall survival between groups (*p* < 0.05). The RRS demonstrated good prognostic discrimination, with a concordance index of 0.74.

A multivariable Cox proportional hazards model incorporating the six selected radiomic features was used to derive a radiomic risk score for each patient (Figure S5). Using the training cohort median as the threshold, the radiomic risk score stratified patients into high risk and low risk groups with significantly different overall survival in both cohorts. In the training cohort, high-risk score patients had significantly worse survival (HR 2.71, 95% CI 1.86–3.96, *p* < 0.001), with median OS of 32.0 months versus 53.0 months in low-risk score patients. In the test cohort, the RRS retained prognostic value (HR 2.01, 95% CI 1.14–3.54, *p* < 0.001), with median OS of 24.0 months in high-risk patients versus 38.0 months in low-risk patients. Multivariable Cox regression confirmed that the RRS provided prognostic information independent of clinical variables (HR 2.91, 95% CI 1.93–4.39, *p* < 0.001) (Table 2; Fig. 4, Supplementary Fig. S3).

**Table 2:** Multivariable Cox regression including radiomics risk score and clinical covariates in the training cohort.

Covariate	Hazard ratio (95% confidence interval)	*p*-Value
Synchronous presentation	0.80 (0.63–1.00)	0.0529
Left-sided primary	1.13 (0.87–1.47)	0.354
Primary node positive	1.25 (0.99–1.50)	0.084
Lung Metastases	1.70 (1.30–2.20)	< 0.001
Bilobar Metastases	1.11 (0.89–1.40)	0.351
Age (per year)	1.02 (1.01 to 1.03)	< 0.001
Number of metastases (per lesion)	1.05 (1.02 to 1.09)	0.007
Largest size (Fong)	1.28 (0.99–1.65)	0.0615
Neoadjuvant chemotherapy	1.54 (1.19–2.00)	< 0.001
R1 Margin	2.22 (1.80–2.73)	< 0.001
Radiomics Risk Score	2.91 (1.93–4.39)	< 0.001

A comparable risk score was also constructed using clinical variables alone. This clinical risk score stratified patients into high- and low-risk groups with significantly different overall survival in both cohorts (Supplementary Fig. S5). The concordance index for the clinical risk score was 0.69, compared with 0.74 for the radiomic risk score.

## Discussion

In this multicentre retrospective study, we demonstrate that radiomic features derived from preoperative portal venous CT imaging provide prognostic information for patients undergoing resection of colorectal liver metastases. Two complementary analytical approaches were used to evaluate the clinical utility of radiomics. First, binary classification models assessed discrimination for fixed-horizon outcomes. Second, a radiomic risk score (RRS) was derived using a time-to-event framework and evaluated using survival analysis. These approaches address distinct clinical questions and provide complementary insights into the role of radiomics in prognostic modelling.

The AUC-based analyses examined whether radiomics improved dichotomous risk classification at predefined timepoints. In this setting, radiomics did not outperform established clinical variables for prediction of 3-year mortality, although it demonstrated numerically better performance for prediction of 2-year mortality. Combined clinical-radiomic models achieved the highest overall performance. Importantly, formal comparison using the DeLong test demonstrated no statistically significant difference between radiomics and clinical only models at either timepoint, consistent with overlapping confidence intervals. By contrast, the RRS, derived using a time-to-event framework, enabled robust survival stratification and remained independently associated with overall survival (Table 2). These findings suggest that radiomics and clinical variables provide complementary rather than interchangeable prognostic information. Whereas clinical variables capture established determinants of disease burden and progression, radiomics appears to capture additional imaging-derived phenotypic information that contributes to survival stratification. This complementary value is further supported by the finding that models combining radiomics with clinical variables demonstrated the best overall performance.

From a clinical perspective, radiomics can be used as an adjunct preoperative decision support tool within the multidisciplinary team setting. Preoperative CT imaging is already universally available in patients with resectable colorectal liver metastases, and automated extraction of a radiomic risk score could be performed without additional imaging, cost, or patient burden. Presentation of a survival risk category alongside standard clinicopathological variables may help refine treatment selection in scenarios where prognostic uncertainty influences management, such as in elderly patients, those with borderline functional reserve, or cases where the relative benefits of resection versus ablation, stereotactic radiotherapy, or systemic therapy are closely balanced. In this context, radiomics is not intended to replace established clinical judgement, but rather to augment existing prognostic assessment by providing complementary imaging-derived information to support shared decision-making, treatment individualisation, and informed consent prior to intervention.

A key strength of this study is the inclusion of imaging data from two centres with differing patient populations, surgical practices, and imaging environments. Many prior radiomics studies in colorectal liver metastases have been limited to small single centre cohorts, often incorporating high dimensional feature sets. Such approaches are vulnerable to overfitting and optimistic performance estimates^25,35,36^. By applying stringent feature reduction and evaluating performance across centres, our findings provide a more realistic assessment of the true prognostic capability of CT based radiomics.

Existing prognostic approaches in CRLM are based largely on clinicopathological variables. The Fong Clinical Risk Score demonstrates only modest performance in contemporary cohorts, with limited ability to discriminate outcomes, particularly as surgical practice has moved towards functional liver remnant estimation rather than tumour burden indices^11,37,38^. Molecular features such as KRAS mutation status are consistently associated with worse outcomes after liver resection, and incorporating KRAS status into clinical scores improves accuracy^38,39^. However, in the present study KRAS mutation status could not be included due to missing data in a substantial proportion of patients, reflecting a real world scenario in which molecular profiling is frequently unavailable to the multidisciplinary team despite guideline (NICE/NCCN) recommendations for patients with metastatic colorectal cancer. Notably, emerging evidence suggests that radiomic features may act as non-invasive surrogates of tumour biology, including molecular alterations such as KRAS mutation status, raising the possibility that radiomics may partially capture underlying genomic phenotypes^40,41^.

In addition, several clinically relevant variables, including adjuvant systemic therapy, radiologic and pathological response to neoadjuvant treatment, and more detailed characterisation of extrahepatic disease, were not consistently available across both centres in a sufficiently complete and standardised manner to permit robust pooled analysis. While this reflects real-world data availability, it may have limited the comprehensiveness of the clinical model. Resection margin status (R1) was included within the clinical model as a surrogate marker of tumour biology (aggressiveness) and technical resectability (operative complexity); however, this variable is determined postoperatively and may limit strict preoperative applicability. Histopathological features such as growth pattern, including desmoplastic and replacement phenotypes, carry strong prognostic significance but are not routinely available^42^. Serum carcinoembryonic antigen (CEA) level similarly provides prognostic information, yet is not universally expressed in colorectal cancer, limiting its reliability as a standalone biomarker^43^. In contrast, radiomics offers a non-invasive approach that is available preoperatively using routinely acquired imaging. Radiomic signatures have demonstrated predictive performance comparable to or exceeding KRAS mutation status for response to anti EGFR therapy in colorectal cancer, highlighting their potential role in complementing established biomarkers.^44^. Consistent with this, our study showed that radiomics features alone achieved acceptable discrimination for 2-year and 3-year postoperative mortality, supporting their utility in preoperative risk stratification when molecular or histopathological data are unavailable.

Although radiomics provides prognostic information at the time of surgical decision-making, the relationship between specific radiomic features and underlying biological processes remains incompletely understood. The features most predictive of outcome in our analysis were textural and morphological. Texture features quantify spatial variation in pixel intensity, reflecting biological heterogeneity. Such heterogeneity potentially corresponds to differences in necrosis, fibrosis, inflammation or vascularity, all biological features influencing prognosis in cancer^45^. Morphological features capture size, shape, and structural characteristics, also linked to outcome^46^. Given the established association between intra-tumoural heterogeneity and poor prognosis, it is plausible that radiomics is detecting biological heterogeneity as a marker of aggressive CRLM behaviour^47^. Histological growth pattern strongly influences prognosis and therapy response in CRLM, and preliminary studies suggest MRI and CT radiomics can distinguish them, albeit with modest accuracy^48–52^. Establishing causal links between radiomic features, biological features, and clinical outcomes is thus an essential next step to improving the interpretability of future radiomics studies in the setting of CRLM.

We also demonstrate that radiomic features derived from the background liver contribute to prognostic performance. This suggests that radiomics may capture microenvironmental factors such as steatosis, inflammation, or fibrosis, which may influence metastatic progression^53–58^. To our knowledge, this has not been systematically explored in prior CT radiomics studies of CRLM and represents a novel contribution of the present work. Future work should compare radiomics signatures with histopathology of the background liver to determine whether distinct hepatic environments can be identified and potentially targeted for surveillance or therapy.

We recognise that portal-venous CT alone may not provide the most comprehensive assessment of CRLM biology. Single-phase imaging captures only part of tumour behaviour and biology is likely better reflected in multimodal datasets incorporating contrast-enhanced MRI and/or FDG-PET^59,60^. While multimodal radiomics may improve performance, such approaches are resource-intensive and may limit clinical applicability^12^. Furthermore, deep learning radiomics models have been developed using non-contrast CT alone, indicating that modality choice should be weighed against study design and feasibility^17^.

Despite our encouraging results, applications of radiomics in clinical practice requires several challenges to be addressed. Differences in scanners, imaging protocols, and patient populations can affect feature stability, meaning models developed in one centre may not perform well elsewhere^61^. Future efforts should focus on standardising image acquisition, validating models across multiple hospitals, and simplifying workflows so risk scores are generated automatically and discussed in multidisciplinary team meetings^62^. With these developments, radiomics has the potential to become a practical tool supporting routine clinical decision-making in CRLM care. Our findings suggest that the greatest value of radiomics lies in complementary prognostic modelling and survival risk stratification, rather than in standalone prediction.

## Conclusion

This study contributes to the growing body of evidence supporting the clinical potential of radiomics in CRLM prognostication. Using imaging data from two large UK hepatobiliary centres, we demonstrate that CT-based radiomics was independently associated with overall survival in this cohort. While radiomics does not outperform clinical variables for fixed-horizon prediction, it enables robust survival stratification and augments established prognostic models. Future large-scale prospective studies integrating radiomics with molecular and histopathological data are required to refine prognostic models and support clinical translation. Progress in standardisation, harmonisation, and automation will be essential to realise the full potential of radiomics in personalised surgical oncology.

## Supplementary Information

Below is the link to the electronic supplementary material.

Supplementary Material 1

## Data Availability

The datasets generated and analysed during the current study are not publicly available due to patient confidentiality and institutional governance restrictions but are available from the corresponding author on reasonable request, subject to appropriate approvals.
