# Supplementary material for: CT radiomics for survival risk stratification in resectable colorectal liver metastases: a multi-centre study
Source: Sci Rep. 2026 Jun 24;16:19580. doi: 10.1038/s41598-026-48659-0 (PMC13294494; doi:10.1038/s41598-026-48659-0)
Supplement: Supplementary file 1 — Supplementary Material 1 [file 41598_2026_48659_MOESM1_ESM.docx]

**Supplementary Materials**


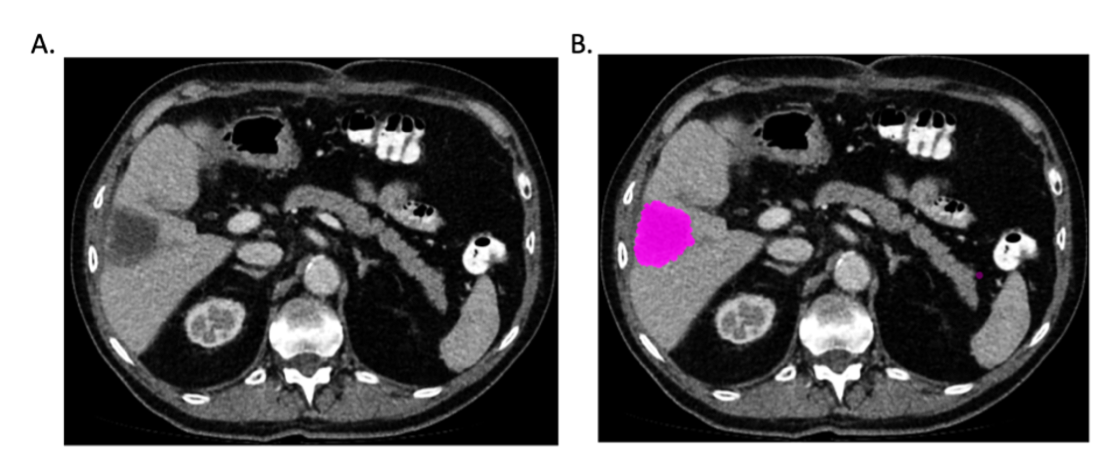


**Figure S1A-B:** Example of region of interest definition in the axial plane. (A) Original portal venous phase CT image. (B) Corresponding image with segmentation mask of a colorectal liver metastasis.


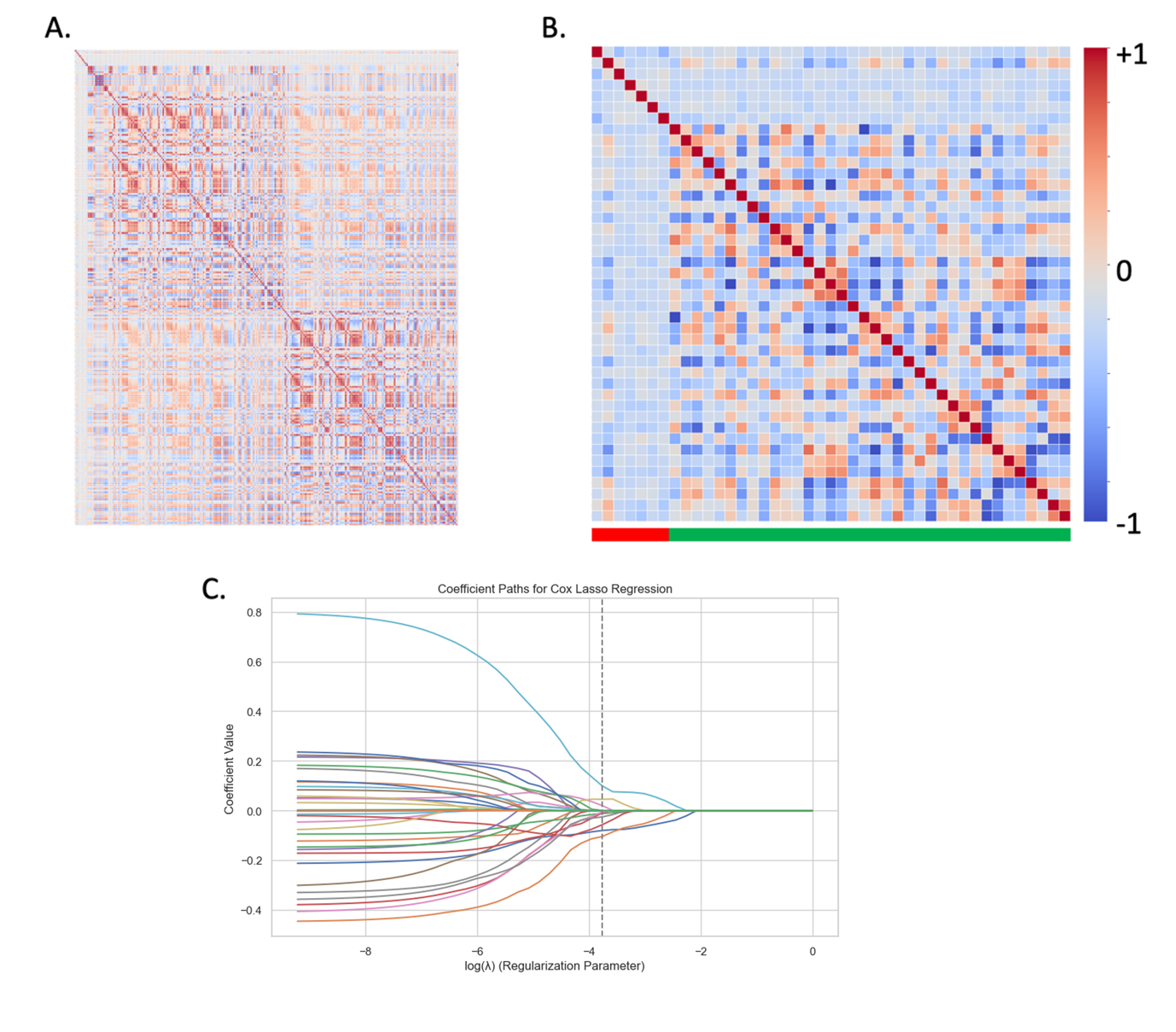


**Figure S2. Radiomics feature selection process using training cohort only. A)** Initial correlation matrix demonstrating correlation between clinical and radiomic features generated from CRLM and background liver. **B)** Following removal of highly correlated features demonstrating absolute Pearson correlation >0.75 showing clinical (red bar) and radiomics features (green bar). **C)** Further feature reduction using L1 cox regularisation, plot of the coefficient path with varying regularisation strength for each specific feature in the training data and the cut-off for further feature exclusion.

a)


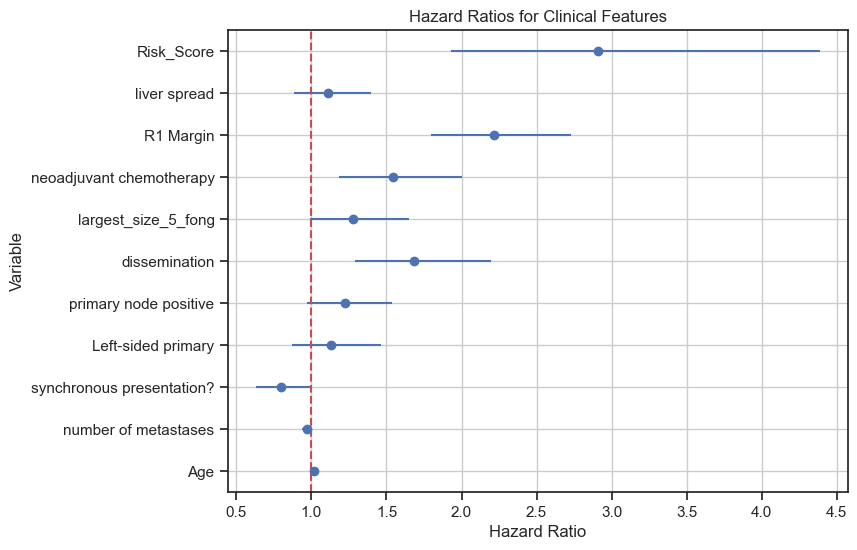


b)


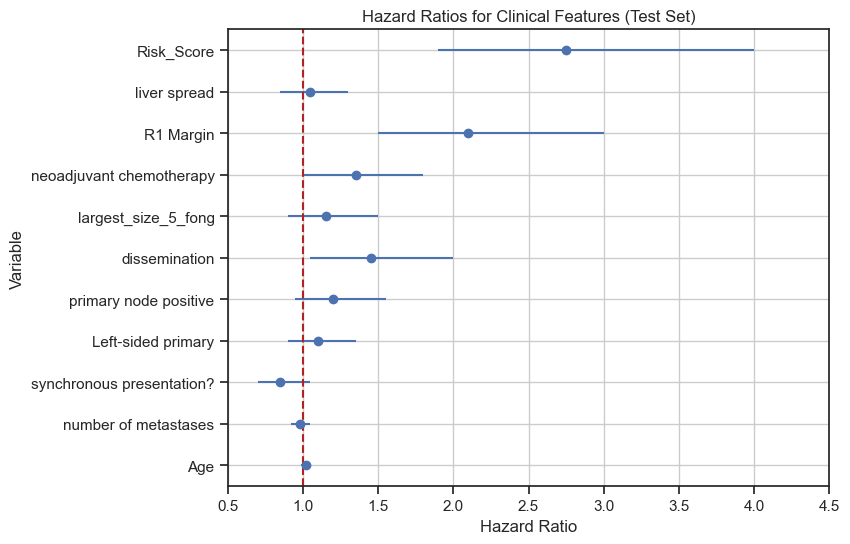


**Figure S3:** Forest plots of hazard ratios in the training set (a) and independent test set (b) for the radiomics risk score and selected clinical covariates. Lung metastases refers to the presence of extrahepatic pulmonary disease (dissemination). Bilobar disease refers to involvement of both hepatic lobes (liver spread). Largest_size_5_fong refers to a maximum tumour diameter >5 cm, as defined in the Fong Clinical Risk Score. R1 +ve margin indicates microscopic tumour involvement at the resection margin. Estimates in the test set are shown for independent validation, with radiomic risk scores calculated using coefficients derived exclusively from the training set.

$$Radiomic Risk score=\sum_{i=1}^{n} \left( {MV Cox Coefficient}_{i} \times{feature value}_{i} \right)$$

The Radiomic Risk Score is calculated as the sum of the products of each selected

radiomic feature’s Cox regression coefficient and its feature value for a given patient.

- Coefficient_i_ :Multivariate Cox Regression Coefficient for the i'th selected feature.
- Feature value_i_: Value of the i’th selected radiomic feature for that sample.
- The summation is performed over all selected radiomic features (n) to generate a total radiomic risk score for the sample.

**Figure S4:** The RRS is derived as a linear combination of selected radiomic features, each weighted by its Cox regression coefficient obtained from the training cohort (Table S2). For each patient, the score is calculated as the sum of each feature value multiplied by its corresponding coefficient. These coefficients are fixed after model training and applied unchanged to the independent test cohort, ensuring consistent and unbiased risk score estimation. The RRS is initially calculated as a continuous variable and subsequently dichotomised using the training-set median for survival stratification. Importantly, the RRS reflects the combined multivariable effect of all features and does not require individual features to be independently statistically significant.

**p<0.05**

**p<0.05**


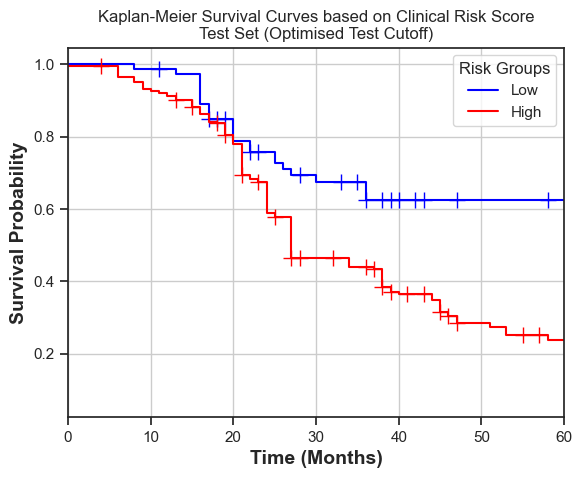

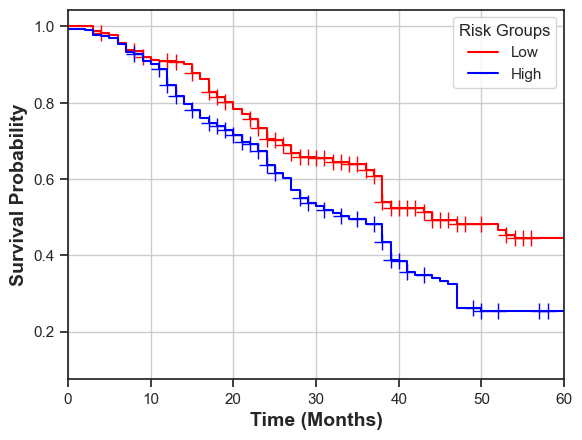


**Figure S5:** Kaplan–Meier survival analysis by Clinical Risk Score. A clinical risk score was derived using the same approach as the radiomic risk score (RRS, Figure S4). Patients were stratified into high- and low-risk groups using the median risk score from the training cohort, and this threshold was applied to the independent validation cohort. Kaplan–Meier curves are shown for (i) the training set and (ii) the validation set. In both cohorts, the log-rank test demonstrated a statistically significant difference in overall survival between risk groups (p < 0.05). The clinical risk score demonstrated a concordance index (C-index) of 0.69, compared with 0.74 for the radiomic risk score, indicating improved prognostic discrimination with the RRS.


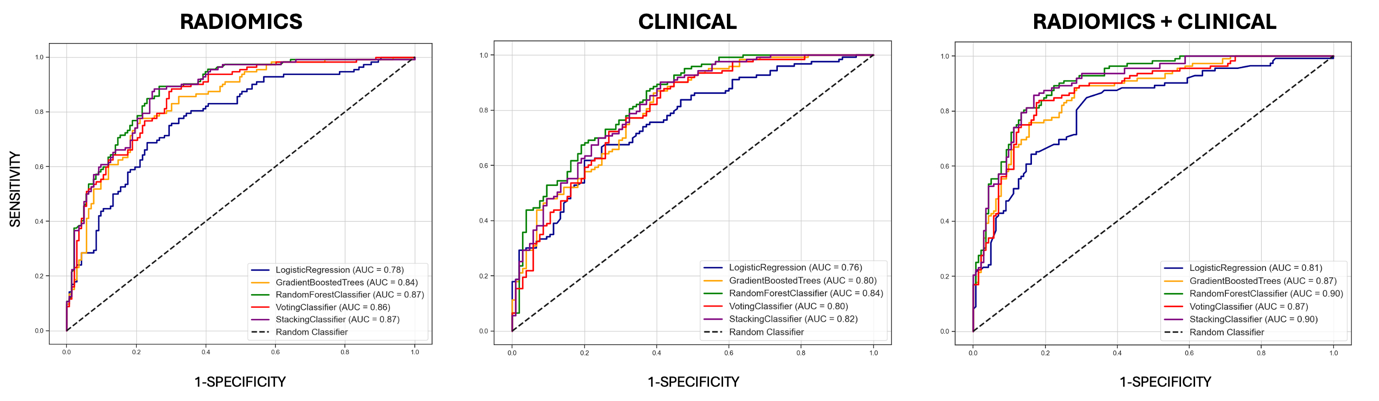


**Figure S6.** Receiver operating characteristic (ROC) curves for prediction of 2-year mortality using radiomics, clinical, and combined radiomics-clinical models. Performance of multiple machine learning classifiers is shown, including logistic regression, gradient boosting, random forest, voting, and stacking classifiers. Radiomics-based models demonstrated moderate-to-good discrimination, while clinical models showed comparable performance. Combined radiomics-clinical models achieved the highest discrimination across classifiers (AUC up to 0.90), indicating complementary prognostic value of imaging-derived and clinical features. The dashed diagonal line represents random classifier performance.


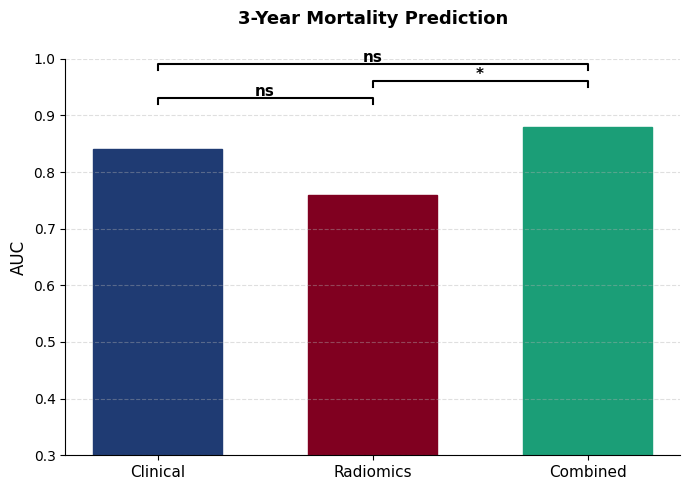

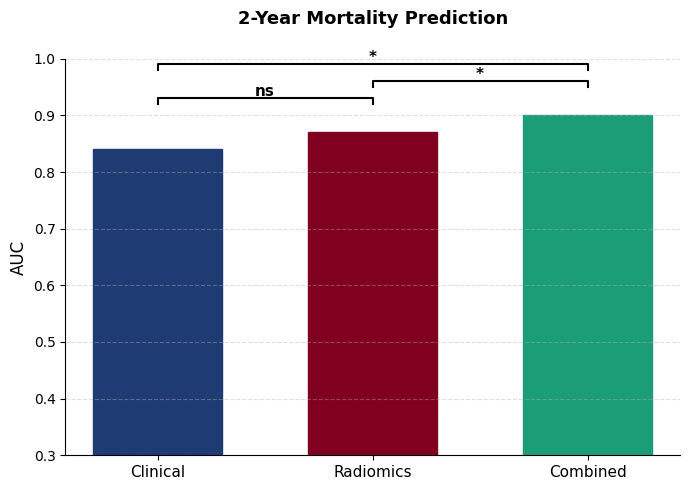


**Figure S7.** Comparison of model discrimination for prediction of 2-year and 3-year mortality using clinical, radiomic, and combined clinical–radiomic models. Bar charts display area under the receiver operating characteristic curve (AUC) for the best performing model in each group. Combined models demonstrated the highest overall performance at both timepoints. Formal comparison using the DeLong test showed no statistically significant difference between clinical and radiomic models, while combined models demonstrated improved discrimination.

**Table S1:** Machine Learning model performances using radiomics, clinical, and combination of both for prediction of a) mortality at 3 years on the test data with 95% confidence intervals (CIs) in independent test set b) mortality at 2 years on the test data with 95% confidence intervals (CIs) in independent test set

1. Mortality at 3 years

|  |  |  | **Machine Learning Models** | | | |
| --- | --- | --- | --- | --- | --- | --- |
| **Features** | **Metric** | **Logistic Regression** | **Gradient Boosting** | **Random Forest** | **Voting Classifier** | **Stacking Classifier** |
| **Clinical & Radiomics** | Accuracy | 0.74 (0.67–0.82) | 0.73 (0.68–0.79) | 0.75 (0.69–0.80), | 0.73 (0.67–0.79) | 0.74 (0.68–0.79) |
|  | AUC | 0.82 (0.76–0.88) | 0.87 (0.82–0.92) | 0.88 (0.83–0.93) | 0.88 (0.83–0.93) | 0.88 (0.83–0.93) |
|  | F1 Score | 0.72 (0.65–0.80) | 0.81 (0.75–0.86) | 0.83 (0.77–0.88) | 0.82 (0.76–0.87) | 0.82 (0.76–0.87) |
| **Clinical** | Accuracy | 0.68 (0.61–0.73) | 0.71 (0.64–0.75) | 0.69 (0.66–0.77) | 0.69 (0.64–0.76) | 0.71 (0.66–0.77) |
|  | AUC | 0.76 (0.66–0.80) | 0.79 (0.72–0.85) | 0.84 (0.78–0.90) | 0.82 (0.75–0.88) | 0.84 (0.78–0.90) |
|  | F1 Score | 0.69 (0.60–0.77) | 0.72 (0.66–0.78) | 0.76 (0.71–0.82) | 0.74 (0.69–0.80) | 0.76 (0.71–0.82) |
| **Radiomics** | Accuracy | 0.68 (0.59–0.71) | 0.73 (0.66–0.81) | 0.73 (0.65–0.81) | 0.71 (0.64–0.79) | 0.73 (0.66–0.81) |
|  | AUC | 0.71 (0.61–0.81) | 0.77 (0.68–0.85) | 0.76 (0.68–0.80) | 0.75 (0.66–0.83) | 0.76 (0.71–0.79) |
|  | F1 Score | 0.66 (0.58–0.74) | 0.72 (0.65–0.79) | 0.71 (0.65–0.78) | 0.70 (0.64–0.76) | 0.71 (0.66–0.78) |

1. Mortality at 2 years

|  |  |  | **Machine Learning Models** | | | |
| --- | --- | --- | --- | --- | --- | --- |
| **Features** | **Metric** | **Logistic Regression** | **Gradient Boosting** | **Random Forest** | **Voting Classifier** | **Stacking Classifier** |
| **Clinical & Radiomics** | Accuracy | 0.76 (0.70–0.83) | 0.82 (0.76–0.88) | 0.84 (0.79–0.89) | 0.83 (0.78–0.89) | 0.84 (0.79–0.89) |
|  | AUC | 0.81 (0.75–0.87) | 0.87 (0.82–0.92) | 0.90 (0.85–0.94) | 0.87 (0.82–0.92) | 0.90 (0.87–0.92) |
|  | F1 Score | 0.74 (0.68–0.80) | 0.80 (0.74–0.86) | 0.83 (0.77–0.88) | 0.82 (0.76–0.87) | 0.83 (0.77–0.88) |
| **Clinical** | Accuracy | 0.72 (0.65–0.78) | 0.75 (0.69–0.81) | 0.79 (0.73–0.85) | 0.77 (0.71–0.83) | 0.79 (0.73–0.85) |
|  | AUC | 0.76 (0.69–0.83) | 0.80 (0.73–0.86) | 0.84 (0.78–0.90) | 0.80 (0.73–0.86) | 0.82 (0.76–0.88) |
|  | F1 Score | 0.71 (0.64–0.77) | 0.74 (0.68–0.80) | 0.77 (0.71–0.83) | 0.75 (0.69–0.81) | 0.77 (0.71–0.83) |
| **Radiomics** | Accuracy | 0.71 (0.63–0.78) | 0.77 (0.70–0.84) | 0.76 (0.69–0.83) | 0.75 (0.68–0.82) | 0.76 (0.69–0.83) |
|  | AUC | 0.78 (0.71–0.85) | 0.84 (0.78–0.90) | 0.87 (0.82–0.92) | 0.86 (0.80–0.91) | 0.87 (0.82–0.92) |
|  | F1 Score | 0.70 (0.62–0.77) | 0.76 (0.69–0.82) | 0.75 (0.69–0.81) | 0.74 (0.68–0.80) | 0.75 (0.69–0.81) |

AUC (Area Under the Curve) is a discriminatory metric used to evaluate the performance of a binary classifier. It represents the area under the ROC (Receiver Operating Characteristic) curve, which plots the True Positive Rate (Sensitivity) against the False Positive Rate (1 - Specificity) across different threshold levels. Accuracy is the proportion of correct predictions (both true positives and true negatives) out of the total predictions. The F1 score is the harmonic mean of precision and recall. It balances the trade-off between precision and recall. Confidence intervals were derived using bootstrap resampling of the independent test cohort.

**Table S2:** Multivariate Cox Regression using selected radiomic features from training cohort with corresponding β Coefficient, Hazard Ratio, 95% Confidence Interval (CI) and p-Values.

| **Feature** | **Category** | **β Coefficient** | **Hazard Ratio** | **95% CI** | **p-Value** |
| --- | --- | --- | --- | --- | --- |
| MET_MORPHOLOGICAL_Compactness2(IBSI:BQWJ) | Morphological | -0.145 | 0.865 | (0.753 - 0.994) | <0.05 |
| MET_INTENSITY-HISTOGRAM_IntensityHistogramMaximumGreyLevel(HU)IBSI:3NCY | First-order (intensity histogram) | 0.110 | 1.116 | (1.002 -1.245) | <0.05 |
| MET_GLCM_Correlation(IBSI:NI2N) | Texture (GLCM) | -0.094 | 0.910 | (0.823 - 0.998) | <0.05 |
| NOR_GLRLM_ShortRunHighGreyLevelEmphasis(IBSI:GD3A) | Texture (GLRLM) | 0.003 | 1.003 | (0.832 -1.209) | 0.14 |
| NOR_NGTDM_Strength(IBSI:1X9X) | Texture (NGTDM) | -0.127 | 0.881 | (0.832 -1.208) | 0.091 |
| NOR_GLSZM_ZoneSizeEntropy(IBSI:GU8N) | Texture (GLSZM) | -0.110 | 0.896 | (0.810 - 0.993) | <0.05 |

**Table S3.** Radiomic features included in the Radiomic Risk Score and their possible interpretation

| **Feature name (IBSI)** | **Feature class** | **Region of interest** | **Description** | **Potential biological interpretation** |
| --- | --- | --- | --- | --- |
| MET_MORPHOLOGICAL_Compactness2 (BQWJ) | Morphological | Tumour | Quantifies how compact the tumour shape is relative to an ideal sphere | Irregular tumour morphology may reflect invasive growth and aggressive behaviour |
| MET_INTENSITY_HISTOGRAM_MaximumGreyLevel (3NCY) | First order intensity | Tumour | Maximum voxel intensity within the tumour region | May reflect focal regions of high cellularity, fibrosis, or altered vascular perfusion |
| MET_GLCM_Correlation (NI2N) | Texture | Tumour | Measures linear dependency of grey level intensities between neighbouring voxels | Reflects spatial organisation and heterogeneity within tumour tissue |
| NOR_GLRLM_ShortRunHighGreyLevelEmphasis (GD3A) | Texture | Background liver | Emphasises short contiguous runs of high intensity voxels | May capture fine scale heterogeneity related to fibrosis, inflammation, or steatosis |
| NOR_NGTDM_Strength (1X9X) | Texture | Background liver | Quantifies perceptible intensity differences between a voxel and its neighbourhood | Reflects uniformity of the liver microenvironment, potentially influencing metastatic permissiveness |
| NOR_GLSZM_ZoneSizeEntropy (GU8N) | Texture | Background liver | Measures variability in size distribution of homogeneous intensity zones | Indicates heterogeneity of liver tissue architecture and extracellular matrix organisation |

*MET denotes tumour derived features and NOR denotes background liver derived features. All features are defined according to Image Biomarker Standardisation Initiative guidelines. Biological interpretations are hypothesised associations and do not imply direct mechanistic causation.*

**Table S4.** Summary of extracted radiomic features by feature class and region of interest

| **Feature class** | **Feature type** | **Description** |
| --- | --- | --- |
| Morphological | Shape and size descriptors | Quantify three dimensional tumour or parenchymal geometry including volume, surface area, compactness, sphericity, and spatial distribution |
| First order | Intensity based statistics | Describe voxel intensity distribution within the region of interest including measures of central tendency and dispersion |
| Second order | Texture features | Quantify spatial relationships and heterogeneity using GLCM, GLRLM, GLSZM, and NGTDM matrices |
|  |  |  |

*Radiomic feature sets were extracted from tumour regions of interest and matched background liver regions for each patient. All features were defined according to Image Biomarker Standardisation Initiative standards and extracted using LIFEx software. Feature reduction and selection procedures are described in the Methods.*
